# Isolation and characterization of bacteriophages against virulent *Aeromonas hydrophila*

**DOI:** 10.1186/s12866-020-01811-w

**Published:** 2020-06-01

**Authors:** Jin Liu, Shanshan Gao, Yuhao Dong, Chengping Lu, Yongjie Liu

**Affiliations:** 1 Joint International Research Laboratory of Animal Health and Food Safety, College of Veterinary Medicine, Nanjing Agricultural University, Nanjing, 210095 China; 2 Sucheng District Animal Husbandry and Veterinary Station, Suqian, 223800 China

**Keywords:** *Aeromonas hydrophila*, Bacteriophage, Biological characteristics, Biofilm, Phage therapy

## Abstract

**Background:**

*Aeromonas hydrophila* is an important water-borne pathogen that leads to a great economic loss in aquaculture. Along with the abuse of antibiotics, drug-resistant strains rise rapidly. In addition, the biofilms formed by this bacterium limited the antibacterial effect of antibiotics. Bacteriophages have been attracting increasing attention as a potential alternative to antibiotics against bacterial infections.

**Results:**

Five phages against pathogenic *A. hydrophila*, named N21, W3, G65, Y71 and Y81, were isolated. Morphological analysis by transmission electron microscopy revealed that phages N21, W3 and G65 belong to the family *Myoviridae*, while Y71 and Y81 belong to the *Podoviridae*. These phages were found to have broad host spectra, short latent periods and normal burst sizes. They were sensitive to high temperature but had a wide adaptability to the pH. In addition, the phages G65 and Y81 showed considerable bacterial killing effect and potential in preventing formation of *A. hydrophila* biofilm; and the phages G65, W3 and N21 were able to scavenge mature biofilm effectively. Phage treatments applied to the pathogenic *A. hydrophila* in mice model resulted in a significantly decreased bacterial loads in tissues.

**Conclusions:**

Five *A. hydrophila* phages were isolated with broad host ranges, low latent periods, and wide pH and thermal tolerance. And the phages exhibited varying abilities in controlling *A. hydrophila* infection. This work presents promising data supporting the future use of phage therapy.

## Background

*Aeromonas hydrophila* is a Gram-negative, rod-shaped bacterium that is ubiquitous found in natural aquatic environments. The bacterium is predominantly pathogenic to poikilothermy animals, including fish, turtles, snakes and amphibians [1]. It is responsible for the large-scale outbreak of fish hemorrhagic septicemia, leading to severe economic losses to aquaculture industry worldwide [2]. The bacterium is also an important pathogen that infects humans and other mammals, causing gastroenteritis and various systemic infections. *A.hydrophila* is a very typical pathogen of human-animal-fish comorbidity [1].

In general, the prevention and treatment of diseases depend mainly on extensive application of antimicrobial agents [3, 4]. Antibiotics not only kill the target bacteria, but might also disrupt the host’s normal flora and the ecological balance of the water environment [5]. With the frequent use of antibacterial drugs in aquaculture, multidrug resistance (MDR) of *A. hydrophila* strains are emerging; and drug residues in aquaculture products and the environment are getting worse [6–8]. Vivekanandhan et al. [9] indicated that 99% of *A. hydrophila* strains isolated from fish and prawns were resistant to methicillin, rifampicin, bacitracin, and novobiocin. Recently, De Silva et al. [10] tested antibiotic resistance of 32 strains of *Aeromonas*, and found that all the isolates were multidrug resistant and 100% resistant to ampicillin, colistin, vancomycin and cephalothin. In addition, each of the 43 *Aeromonas* strains isolated by Hossain et al. [11] from 46 zebrafish was resistant to at least four antibiotics. MDR strains can reproduce and pass its resistance on, creating many more antibiotic resistant bacteria [12, 13]. Additionally, many bacterial infections are due to biofilm-embedded bacteria [14]. *A. hydrophila* is capable of forming biofilms on host tissues, as well as multiple biotic and abiotic aquaculture substrates [15]. Bacteria within a biofilm are more resistant to antibiotics compared to those in the planktonic style, since biofilm structure provides a reduced penetration of antibacterial compounds into the biofilm [16, 17], which make treatment of bacterial infections face a serious challenge. Thus, new antimicrobial therapies are urgent to be developed.

Bacteriophages, naturally-occurring bacterial viruses that can kill specific bacteria with no chemical residues and does not affect other flora, are one of the potential alternatives. A small dose of phage can achieve a good therapeutic effect as phage proliferation via auto “dosing” results in great bacterial killing [18]. One previous study from Nishikawa et al. [19] reported that phage KEP10 intraperitoneally injected into mice could immediately spread to all organs examined and maintained a high titer; and treatment of the phage into the peritoneal cavity significantly decreased the mortality of mice inoculated transurethrally with a multidrug-resistant strain of uropathogenic *Escherichia coli* (UPEC). Another study indicated that infection of methicillin-resistant *Staphylococcus aureus* with subsequent administration of purified phage phi MR11 effectively suppressed *S.aureus*-induced bacteremia and lethality in mice [20]. Phage therapy has also shown its efficacy in several cases of *Aeromonas* diseases. Jun et al. [21] reported that phages pAh1-C and pAh6-C could provide protective effects against mass mortality of the cyprinid loach caused by *A. hydrophila*. Additionally, therapeutic treatments of *A. hydrophila*-phage 2 and *A. hydrophila*-phage 5 applied to the catfish during bacterial infection resulted in a significantly enhanced survival of the tested fishes [22]. However, not all phages make for good therapeutics. In general, the narrowness of phage host ranges will limit putative treatment and the lack of phage stability will reduce treatment efficiency. Thus, it is necessary to isolate stable phages with relatively wide host ranges for phage therapy.

In this study, we isolated and characterized five phages specific to *A. hydrophila*. Furthermore, we investigated the efficiency of the phages for biofilm inhibition and removal, and performed a phage therapy experiment in *A. hydrophila*-infected mice.

## Results

### Isolation of *A. hydrophila* phages

**Fig. 1:**
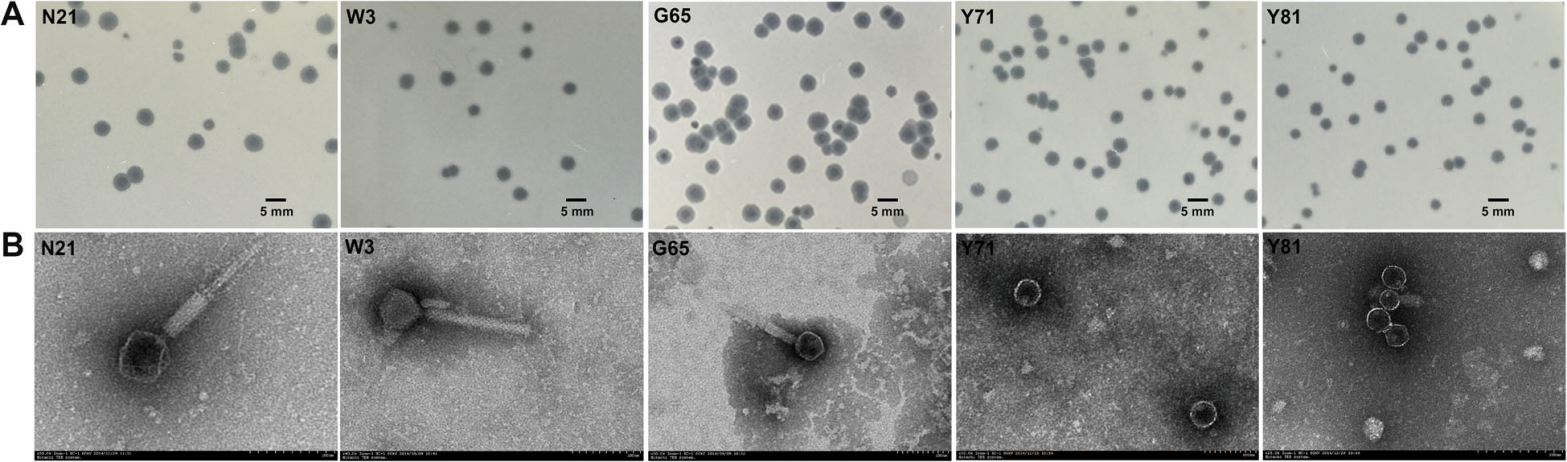
Morphology of phages. **a** Plaque morphology of phages N21, W3, G65, Y71 and Y81 that were plated on 0.7% LB agar overlays with suspensions of *A. hydrophila* and incubated at 28 °C for 16 h. **b** Transmission electron micrographs of phage virions negatively stained with 2% uranyl acetate

Three phages designated as N21, W3 and G65 with *A. hydrophila* NJ-35 as an indicator host, and two phages Y71 and Y81 with *A. hydrophila* XY-16 as an indicator host, were isolated from fish ponds and polluted rivers in Nanjing. Clear plaques appeared after 12 h incubation at 28 °C. As shown in Fig. 1a, plaques of all the five phages were morphologically similar with diameters of 1 mm to 3 mm, and transparent in the middle. The plaque edges of phages W3, Y71 and Y81 were clear with no halo, while those of phages N21 and G65 were blurred with haloes.

### Phage morphology

Purified phages were examined by transmission electron microscopy (TEM) and classifed based on the criteria proposed by Ackermann [23]. TEM observation revealed that all the phages (Fig. 1b) had tails and thus belonged to the order *Caudovirales*. Phages N21, W3 and G65 possessed a morphology typical of the *Myoviridae* family, displaying an icosahedral head with the diameter of (62.6 ± 1.9) nm, (64.9 ± 3.2) nm and (58.8 ± 4.1) nm, respectively, a contractile tail with the length of (153.1 ± 6.2) nm, (154.1 ± 1.4) nm and (152.3 ± 9.8) nm with six long fibers, respectively, and collar and base plate structures. Phages Y71 and Y81 morphologically belonged to the *Podoviridae* family, possessing an icosahedral head of (62.8 ± 1.1) nm and (54.8 ± 2.9) nm in diameter, respectively, and a short tail of (22.0 ± 0.2) nm, (20.0 ± 2.2) nm in length, respectively.

### Host ranges

The lytic spectrum of five phages was determined against nine *Aeromonas* species of a total of 205 isolates, including 75 *A. hydrophila*, 85 *A. veronii*, 12 *A. caviae*, 1 *A. bestiarum*, 12 *A. sobria*, 10 *A. media*, 3 *A. salmonicida*, 3 *A. jandaei* and 4 *A. aquariorum* (Table 1), representative of major *Aeromonas* species pathogenic in fish. As shown in Table 2, it was found that 22.67% (*n* = 17), 21.33% (*n* = 16), 21.33% (*n* = 16), 20% (*n* = 15) and 22.67% (*n* = 17) of 75 *A. hydrophila* isolates were susceptible to phages N21, W3, G65, Y71 and Y81. Additionally, all of the phages could infect one strain of *A. caviae*; phages N21, W3 and G65 also showed infectivity to *A. veronii* of 1, 3, and 3 strains, respectively; and phage W3 was able to infect the only *A. bestiarum* isolate tested in this study, showing broad infectivity against phylogenetically distant species in *Aeromonadaceae*.

**Table 1:** *Aeromonas* strains used in this study

*Aeromonas* species	Sources			
	Fish	Shrimp	Crab	Water
***A. hydrophila***	NJ-35, XY-16, XX-58, NJ-34, NJ-28, ML-2, ML-11, ML-15, XH-3, XS-1, XS-6, XS-7, XH-3 NJ-1, XX-11, XX-12, XX-13, XX-14, NJ-28, JH-19, CS-60, J-1, DW-4, ML-9, ML-13, ML-21, XH-5, CH-2, XS-5, XS-10, CH-8, XS-12, XH-5 XX-49, XX-52, JH-17, DW-2, ML-4, ML-16, ML-22, CH-1, CH-6, XS-3, XS-9, XS-11, JD-3, GY-23, DW-3, ML-12, ML-19, ML-23, XH-1, XH-6, CH-3, CH-10, XS-4, JD-4, XH-1, XH-6, XX-62, DW-1, ML-5, ML-14, ML-17, ML-26, CH-4, CH-11, CH-9, XS-2, JD-1, JD-2			ML-30, ZG-22, SQ-11, NJ-3
***A. veronii***	XH-4, XH-5, CH-5, DS-4, DS-6, ML-10, LK-1, ZG-15, ZG-5, ZG-2, GY-37, GY-28, GY-13, ML-10 LK-1, ML-25, CH-7, CH-12, DS-1, ML-3, ZG-12, ZG-11, ZG-10, ZG-6, ZG-4, GY-41, GY-40, DS-7, ML-3, ML-25, GY-36, DS-2, DS-5, ML-24, ZG-17, ZG-13, DS-9, XH-4, ML-24, DS-3, ML-6, ZG-18, ZG-14, GY-36, DS-8, DS-10, LK-16, LK-15, ML-6, LK-3, SQ-4, SQ-2, ZG-16, ZG-9, ZG-7, ZG-3, LK-3	SQ-5, SQ-6, SQ-7, ML-8, WX-1, WX-2, WX-3, WX-4, LK-17	LK-19, LK-21, LK-22	GY-11, GY-32, GY-53, GY-54, GY-58, ZG-19, ZG-21, ZG-23, SQ-8, SQ-9, ZX-4, ML-29, LK-26, XS-13, LK-27, DS-11
*A. caviae*	ML-1, SQ-1, SQ-3, LK-6, LK-10, LK-4, LK-5, XS-8	ZX-2, LK-18	LK-20	ML-27
*A. bestiarum*				NJ-24
*A. sobria*	LK-2, LK-12, CS-40, GY-45, JH-1, ZG-8, GY-4, LK-14	ZX-1		LK-24, ZG-20, SQ-10
*A. media*	NJ-4, NJ-30, NJ-5, NJ-6, NJ-32			NJ-7, NJ-8, NJ-21, NJ-25, NJ-29
*A. salmonicida*				XX-27, XX-28, CS-2
*A. jandaei*	XH-2, ZG-1			LK-23
*A. aquariorum*	ML-18, LK-25, ML-20			ML-28

**Table 2:** The information of the phages isolated in this study

Phages	Sources	Indicator bacteria	Host ranges			
			*A. hydrophila*	*A.caviae*	*A. veronii*	*A. bestiarum*
N21	Pond water polluted by diseased fish	*A. hydrophila* NJ-35	NJ-35, XY-16, J-1, GY-23, NJ-34, XH-3, XH-4, XH-5, XH-6, CH-3, ML-4, ML-5, ML-11, ML-12, ML-23, CH-8, SQ-11	ML-27	CH-7	
W3	Pond water polluted by diseased fish	*A. hydrophila* NJ-35	NJ-35, XY-16, GY-23, NJ-34, XH-3, XH-4, XH-5, XH-6, CH-3, ML-4, ML-5, ML-11, ML-12, ML-23, CH-8, SQ-11	ML-27	GY-40, SQ-7, CH-7	NJ-24
G65	Polluted river	*A. hydrophila* NJ-35	NJ-35, XY-16, GY-23, NJ-34, XH-3, XH-4, XH-5, XH-6, CH-3, ML-4, ML-5, ML-11, ML-12, ML-23, CH-8, SQ-11	ML-27	GY-40, SQ-7, CH-7	
Y71	Pond water polluted by diseased fish	*A. hydrophila* XY-16	NJ-35, XY-16, J-1, GY-23, NJ-34, XH-3, XH-4, XH-5, XH-6, ML-4, ML-5, ML-12, ML-23, CH-3, SQ-11		CH-7	
Y81	Pond water polluted by diseased fish	*A. hydrophila* XY-16	NJ-35, XY-16, J-1, GY-23, NJ-34, XH-3, XH-4, XH-5, XH-6, CH-3, ML-4, ML-5, ML-11, ML-12, ML-23, CH-8, SQ-11		CH-7	

### Multiplicity of infection (MOI)

*A. hydrophila* cultures of exponential growth phase were infected with different amount of phages as designed. The phage titers were measured after incubation for 2 h. The results indicated that the optimal MOIs of phage isolates N21, W3, G65, Y71 and Y81 were 0.01, 1, 0.001, 0.1 and 0.001, respectively, which gave the highest production of phage progeny (Table 3).

**Table 3:** The optimal MOI of phage N21, W3, G65, Y71 and Y81

Bacteria (CFU/mL)	Phages (PFU/mL)	MOI	Titers of phages after 2 h co-culture (PFU/mL)				
			N21	W3	G65	Y71	Y81
10^6^	10^8^	100	1.28 × 10^8^	2.00 × 10^8^	1.72 × 10^7^	4.00 × 10^7^	7.30 × 10^7^
10^7^	10^8^	10	2.50 × 10^8^	2.50 × 10^8^	5.50 × 10^7^	2.54 × 10^8^	3.55 × 10^8^
10^8^	10^8^	1	3.10 × 10^8^	6.03 × 10^8^	5.70 × 10^7^	2.03 × 10^9^	3.58 × 10^8^
10^8^	10^7^	**0.1**	4.10 × 10^8^	**8.00 × 10**^**8**^	1.17 × 10^8^	**2.50 × 10**^**9**^	3.60 × 10^8^
10^8^	10^6^	**0.01**	**8.93 × 10**^**8**^	1.00 × 10^8^	2.16 × 10^8^	1.15 × 10^9^	3.80 × 10^8^
10^8^	10^5^	**0.001**	2.06 × 10^8^	1.00 × 10^8^	**2.31 × 10**^**8**^	1.00 × 10^9^	**4.46 × 10**^**8**^
10^8^	10^4^	0.0001	2.0 × 10^8^	8.00 × 10^7^	2.00 × 10^8^	8.60 × 10^8^	3.76 × 10^8^

### Latent times and phage burst sizes

**Fig. 2:**
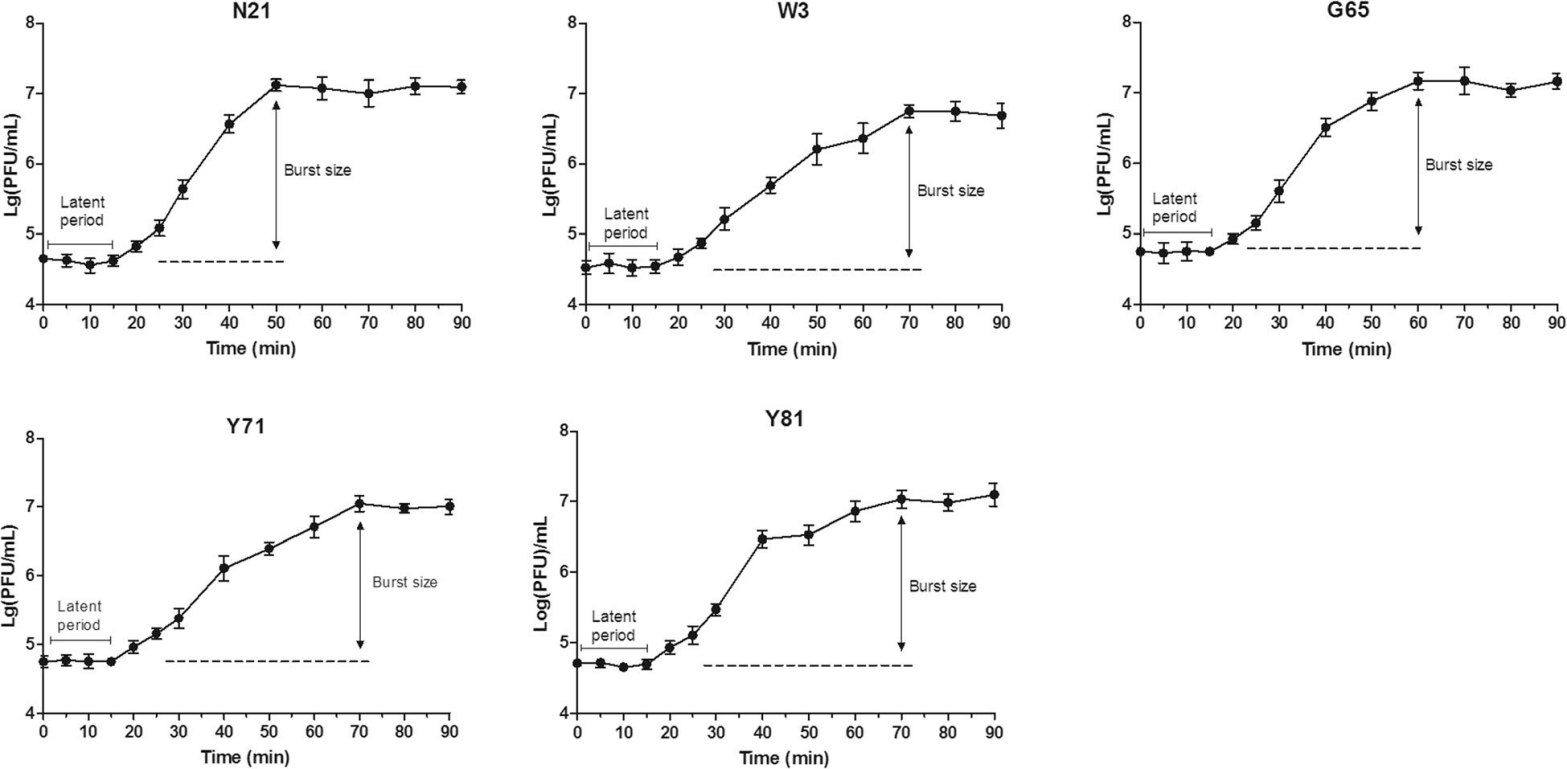
One step growth curves of phages N21, W3, G65, Y71 and Y81. The phage titers were measured by the double-layer agar method. Data are presented as the mean ± standard deviation (SD), and latent periods and burst sizes were inferred from three independent experiments, with each experiment being comprised of three individual measurements

Single step growth experiment was performed to determine the latent time and phage burst size. As shown in Fig. 2, the latent periods of all five phages were found to be about 15 min, and the burst sizes of phages N21, W3, G65, Y71 and Y81 were 316 PFU, 160 PFU, 210 PFU, 200 PFU and 220 PFU per infected host cell, respectively.

### pH and thermal stability

**Fig. 3:**
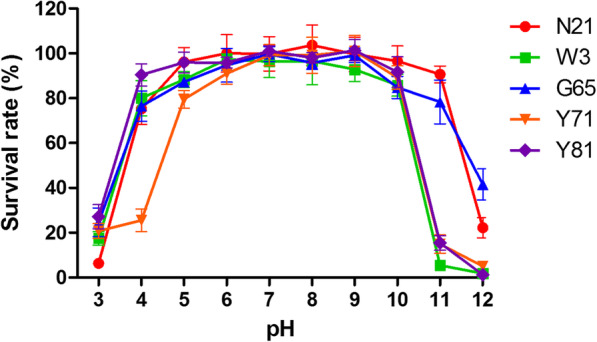
pH stability of phages N21, W3, G65, Y71 and Y81. Phages were incubated for 2 h under different pH values, and the survival rate of phages were shown as mean ± SD from the triplicate experiments

The pH and thermal stabilities of phages were estimated by determining the changes in survival based on the number of plaque-forming units (PFU). As shown in Fig. 3, growth of phage N21 showed no obvious change after 2 h incubation at pH 5.0–11.0, but 75.26% recovery at pH 4.0. The survival of phage W3 could maintain relatively stable at pH 4.0–10.0; very few phages could recovery at pH 3.0 or pH 11.0. Phage Y81 displayed similar pH stability to phage W3. More than 75% phage G65 could survive at pH 4.0–11.0. Phage Y71 showed relatively stable between pH 5.0–10.0. The data suggested that the phages can remain active under a wide range of pH conditions but sensitive to strong acid or alkali.

**Fig. 4:**
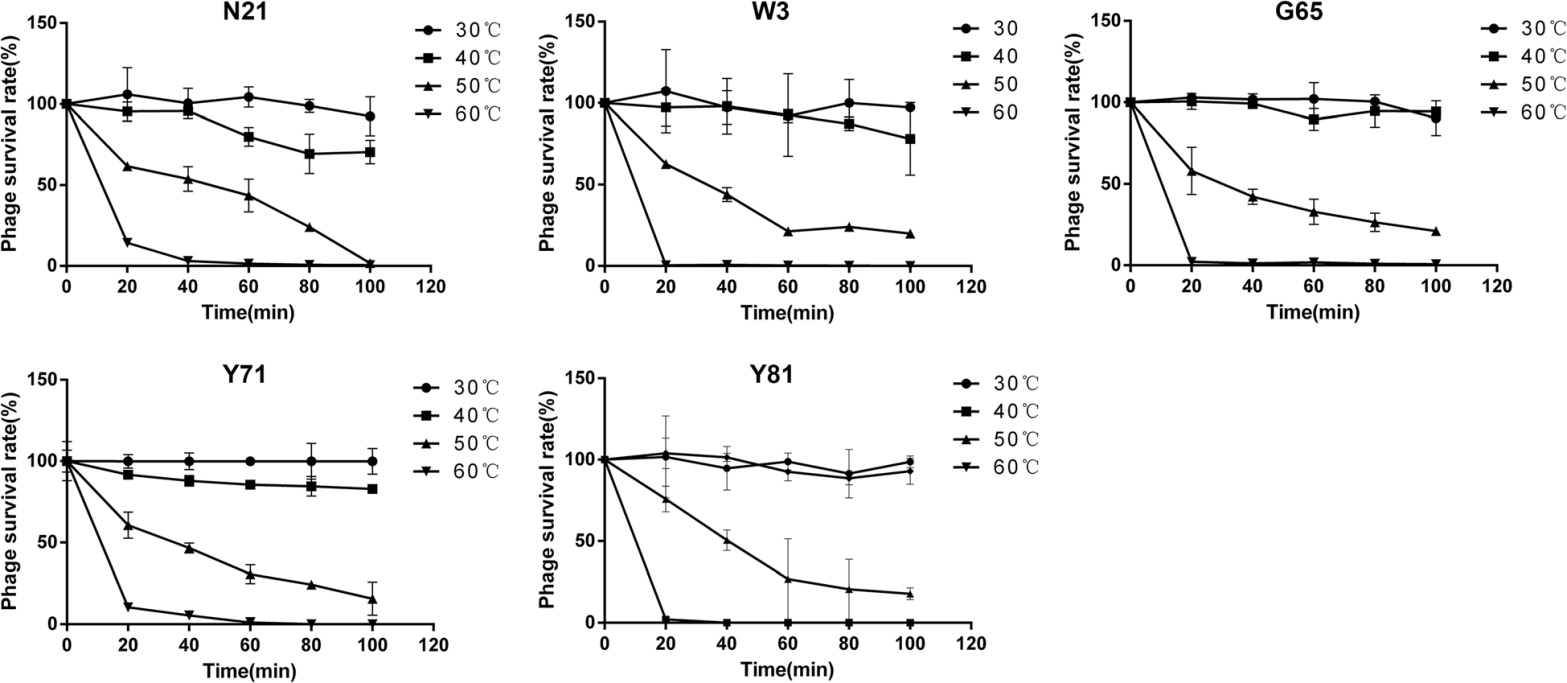
Thermal stability of phages N21, W3, G65, Y71 and Y81. Phages were incubated for 100 min under different temperatures. At an interval of 20 min, the survival rates were calculated by the PFU of viable phages at each time point divided by that at the primary PFU. The test was performed in triplicate

Thermal stability of the isolated phages was assayed at pH 7.0. All of the five phages maintained almost 100% infectivity after cultured at 4 °C or 30 °C for 1 day (data not shown). Figure 4 showed that all the phages remained relatively stable at 30 °C and 40 °C, but sensitive to higher temperatures. No more than 50% phages remained alive after a 40-min incubation at 50 °C. At 60 °C, no more than 1% of phages W3, G65, and Y81 survived for 20 min, and phages N21 and Y71 for 40 min.

### Bacteriolytic activity in vitro

**Fig. 5:**
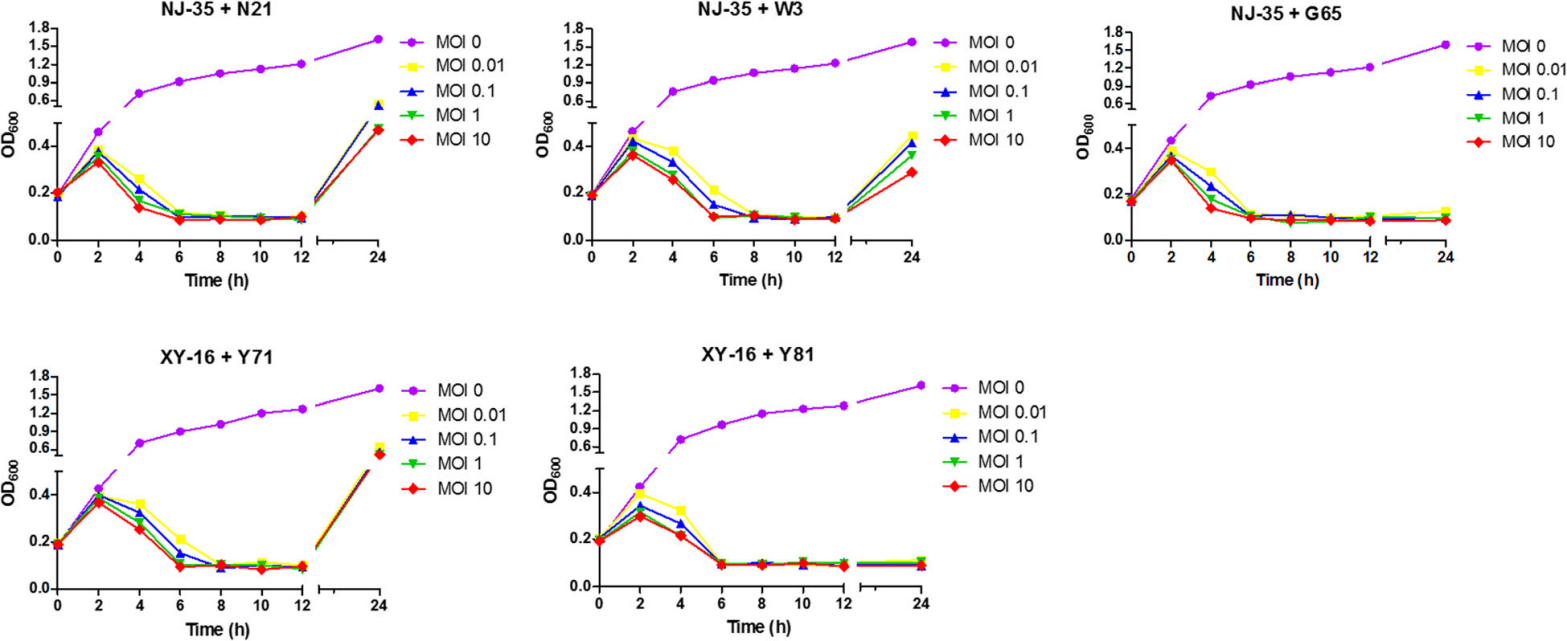
Bactericidal effect of phages in vitro. *A. hydrophila* NJ-35 and XY-16 suspensions (1 × 10^8^ CFU/mL) in exponential phage mixed with phages at different MOIs of 0, 0.01, 0.1, 1 and 10 were incubated at 28 °C for 24 h. The OD_600_ of the culture was measured at each time point during the incubation. The experiment was performed in triplicate

The bacteriolytic activities of five phages were evaluated using *A. hydrophila* strains NJ-35 and XY-16 at different doses of MOIs. As shown in Fig. 5, the absorbance of NJ-35 and XY-16 cultured without phages increased continuously within 24 h, whereas the absorbance of the cultures with the phages increased gradually during the first 2 h, then decreased remarkably in a MOI-dependent manner (2–6 h), and at 6 h, dropped to the minimum at all different MOIs. From 6 h to 24 h, the absorbance of the cultures with phage G65 or Y81 remained stable at its lowest level. However, notably, the absorbance of the cultures began to rise remarkably from 12 h after treatment by phage N21, W3, or Y71.

### Effect of phages on bacterial biofilm formation

**Fig. 6:**
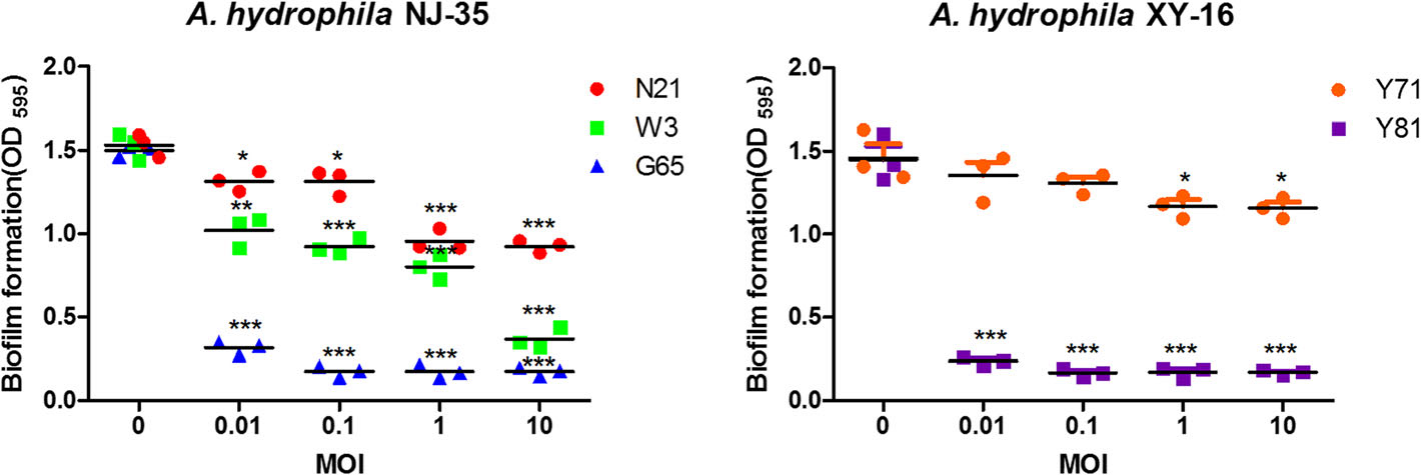
Effect of phages on preventing formation of biofilm. The biofilm formation ability was measured in *A. hydrophila* NJ-35 co-incubated with phage N21, W3 of G65 (A), and *A. hydrophila* XY-16 co-incubated with phage Y71 or Y81 (B) at an MOI of 0.01, 0.1, 1 and 10, using crystal violet staining method. A bacterial culture with no phage served as a control. Data are shown as the mean ± SD from the triplicate experiments. ***P < 0.001, **P < 0.01 or *P < 0.05 indicates a significant difference between this group and the control with no phage treatment

The capability of the phages to prevent biofilm formation of *A. hydrophila* NJ-35 and XY-16 was detected at 24 h after coculture using crystal violet method. As shown in Fig. 6, compared to *A. hydrophila* cultured alone, the bacteria cocultured with phages showed a considerably decreased biofilm formation at each MOI, and the decrease was MOI-dependent. At a MOI of 1.0, all the phages exhibited a significantly inhibited effect on the biofilm formation of their corresponding host strains (*P* < 0.05 or *P* < 0.001). Interestingly, similar to what was observed in bacteriolytic activity, phages G65 and Y81 had stronger abilities to prevent biofilm formation than the other three phages. Very few biofilm could be detected when the *A. hydrophila* was incubated with G65 or Y81 even at a small MOI of 0.01.

### Biofilm clearance ability

**Fig. 7:**
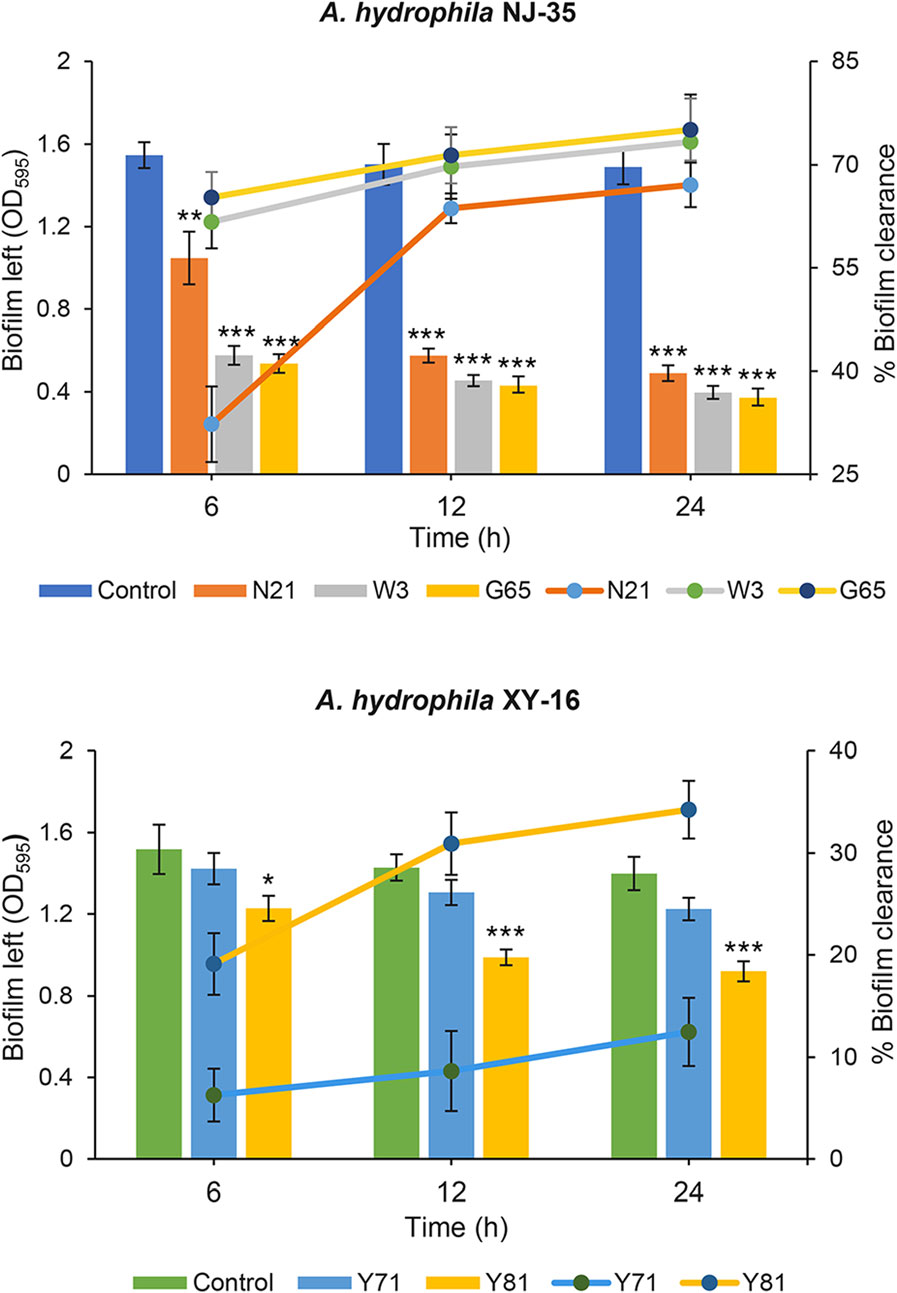
Biofilm clearence ability of phages. Biofilms of *A. hydrophila* NJ-35 formed in 96-well plates for 24 h were treated with phage N21, W3 or G65 at concentrations of 10^8^ PFU/mL, and biofilms of *A. hydrophila* XY-16 formed were treated with phage Y71 of Y81. Biofilms treated with SM buffer served as control. The biofilm left was stained with 1% crystal violet and measured at OD_595_ (bar chart). The percentage of biofilm left was calculated by dividing OD_595_ of phages treated wells and the OD_595_ of controls without phage treatment multiplied by 100. The percentage was subtracted from 100% in order to get the percentage of biofilm clearance (line chart). Data are shown as the mean ± SD from the triplicate experiments. ***P < 0.001, **P < 0.01 or *P < 0.05 indicates a significant difference between this group and the control with no phage treatment

Decrease of mature biofilm formed by *A. hydrophila* strain NJ-35 or XY-16 treated with phages with a titer of 1.0 × 10^8^ PFU/mL was determined. As shown in Fig. 7, phage treatment caused an obvious reduction in biofilm biomass of *A. hydrophila* in a time-dependent manner, as compared with untreated controls, except for the treatment with phage Y71 which resulted in a slight decrease with no statistical difference. Phages G65, W3 and N21 showed a relatively stronger biofilm clearance efficiency after treating for 24 h, with the clearance rates of 75.12, 73.35 and 67.08%, respectively, whereas phages Y81 and Y71 only exhibited biofilm removal of 34.26 and 12.47%, respectively.

### Phage therapy

**Fig. 8:**
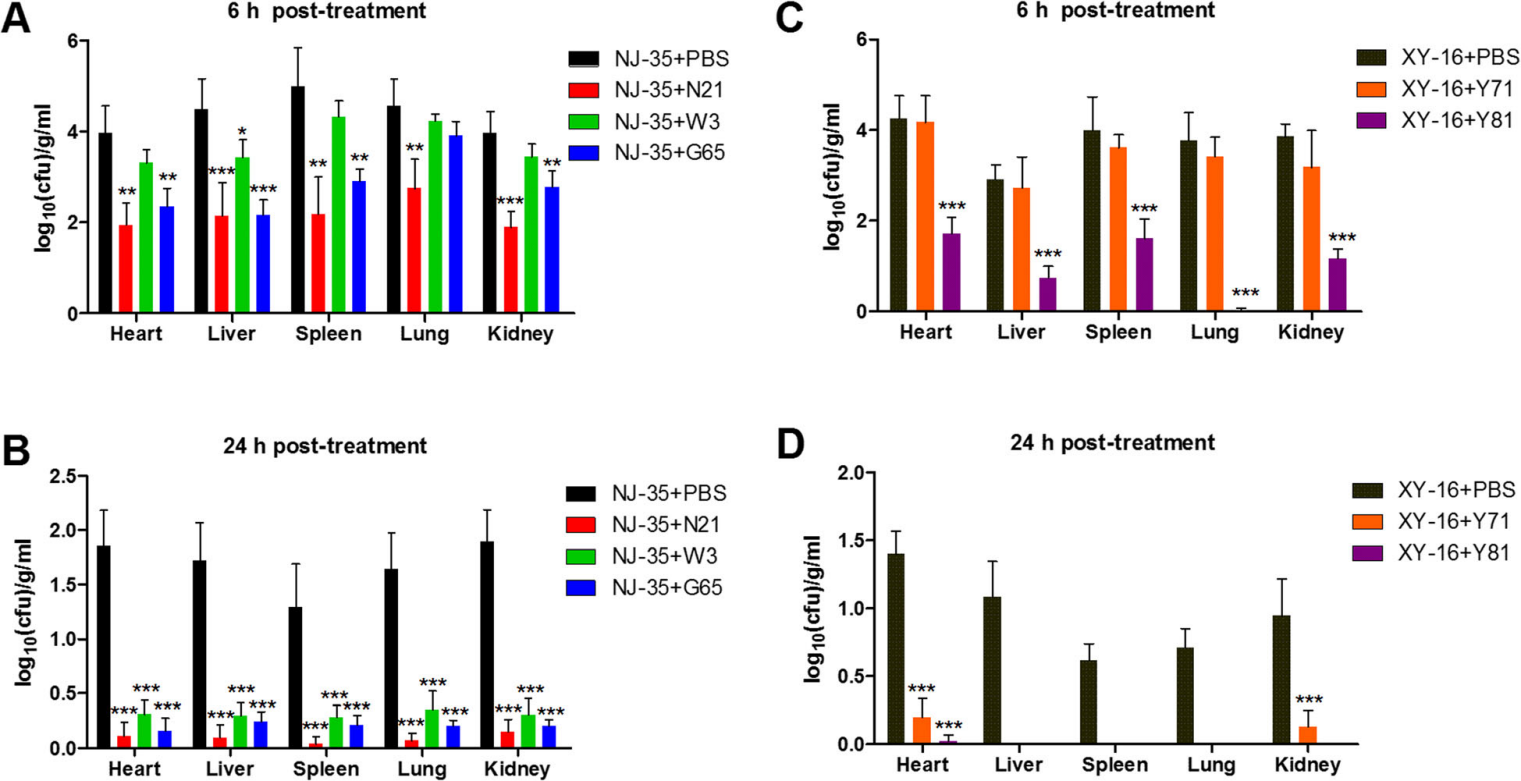
Therapy treatment of phages on *A. hydrophila* infection in mice model. Bacterial loads in the tissues of mice infected with *A. hydrophila* NJ-35 after treating with phages N21, W3 or G65 at the MOI of 1 for 6 h (A) and 24 h (B). Bacterial loads in the tissues of mice infected with *A. hydrophila* XY-16 after treating with phages Y71 or Y81 for 6 h (C) and 24 h (D). Mice treated with PBS after infecting with *A. hydrophila* served as the control. Data are shown as the mean ± SD from five mice in three independ experiments. ***P < 0.001, **P < 0.01 or *P < 0.05 indicates a significant difference between this group and the control with no phage treatment

To corroborate whether the phages can prevent the proliferation of *A. hydrophila* in vivo, we performed an infection and therapy assay in mice. Mice infected with *A. hydrophila* were sacrificed at 6 h and 24 h post-treatment with phages, and hearts, livers, spleens, lungs and kidneys were collected for determination of bacterial loads. As shown in Fig. 8, phages N21 and Y81 treatment lead to significant decrease of the bacterial loads in all tested tissues at 6 h compared to the non-treated controls. After treating for 24 h, the bacterial loads in all tissues of phage-treated groups showed significant decrease compared to the non-treated controls. The results indicated that the phages can act as scavengers to eliminate pathogens in vivo.

## Discussion

Phage taxonomy has progressed from a mainly morphology-based techniques to the modern applications of a holistic approach [24]. Recently, two new phage families *Ackermannviridae* and *Herelleviridae* were established in the order *Caudovirales* based on virion morphology and genome or proteome comparisons [25, 26]. Nevertheless, the tranditional virion structure observation is still the most convenient and important criterion for the bacteriophage classification [27]. In this study, TEM analyses indicated that N21, W3 and G65 virions resembled a novel virulent *Myoviridae* phage, AHP-1, recently identified [28], consisting of an icosahedral head, and a contractile tail with a base plate and six long fibers, but with no “stars” or “prongs” at the base of the tail [29]. Although the virions of *Ackermannviridae* and *Herelleviridae* have been also reported to be of myovirus morphology, the tail spikes are the morphological markers of the family *Ackermannviridae* [29, 30] and the phages of the family *Herelleviridae* always infect members of the phylum *Firmicutes* [31]. Therefore, phages N21, W3 and G65 are actually more closely related to the family *Myoviridae*. At present, most bacteriophages reported against *Aeromonas* spp. have been characterized as tailed phages, and the family *Myoviridae* were found to be the majority [22, 23, 32, 33]. For intance, Ackermann [23] indicated that 33 of 43 *Aeromonas* phages investigated belonged to the *Myoviridae* family. Also, several *Aeromonas* phages belonging to the family *Podoviridae* have been isolated and characterized [34–36]. Phage Y71 and Y81 isolated in this study, possessing an icosahedral head with a short non-contractile tail typical of the *Podoviridae*, were tentatively classified as members of this family.

Desired properties for efficient phage therapy may depend on phage virulence, host range, latent period, obligately lytic property and so on. Broad host range usually exhibites great advantages in combating multiple pathogen infections. In general, phage showed narrow host specificity that only infect its indicator host [37, 38], although there are also some *Aeromonas* bacteriophages that show wide host spectrum [39]. Our study revealed that the phages isolated here had relatively broad spectrum of infectivity against *A. hydrophila* and showed the potential to infect *A. caviae, A. veronii* and *A. bestiarum*. Short latent period and large burst size are essential features for highly effective phages serving as as therapeutic agents [40]. Previous study demonstrated that a T4-like bacteriophage BPA6 infecting *A. hydrophila* had a burst size of 244 phage particles per cell with approximately 10 min of latent period [41]. Similar latent periods and burst sizes of the five phages were observed in our study. Moreover, it is commonly accepted that lytic phages are preferred over temperate phages for phage therapy purpose, although advances in sequencing technologies and synthetic biology are making the temperate phages a great potential for the application of therapy against bacterial infections [42]. Traditionally, the formation of clear plaques has always been used as a presumptive determination of a lytic phage while turbid plaques or especially turbid-centered plaques may imply a temperate phage [43, 44]. All of the five phages isolated in this study form uniform and clear plaques on the *A. hydrophila* strain, suggesting that the phages are most likely to be lytic rather than temperate phages. However, this is not always definitive and further genome-based characterizations of the phages are needed to verify this. Additionally, our phages, being more resistant to alkali than acid condition, showed a broad pH tolerance compared to most isolated phages [38, 45–47]. Besides, the phages were found to be relatively stable up to 40 °C. The high pH and thermal resistance made the phages be potential for possible treatment of *A. hydrophila* infection or contamination in the diverse physicochemical environments.

Expectively, phage therapy in mice model resulted in a decreased bacterial loads in tissues compared to the control groups after treating for 6 h, suggesting a potential role for phages in controlling *A. hydrophila* infection. The therapy effect was found to be time-dependent, and few bacteria were detected after 24 h treatment by any of the phages. It should be noted that for phage N21, W3, or Y71, the bacteriolytic activity in vivo was different from that in vitro, which indicated that bacterial number rapidly decreased to the minimum at 6 h after coculture, but began to rise after 12 h. A reason for this might be the appearance of phage-resistant variants after phage treatment in vitro. Although the emergence of phage-resistant bacteria is typically low as phages are host-specific and self-dosing, it cannot be avoided since it is a natural mechanism of bacteria-virus co-evolution [48]. This phenomenon can occur in phage treatments both in vitro and in vivo [22, 49, 50]. Nevertheless, it has been reported that the appearance of phage-resistant mutants does not affect the effectiveness of therapy in vivo, where the immune system may play a crucial role in the efficiency of phage therapy, especially that neutrophil–phage synergy was essential for the efficient elimination of both phage-sensitive and emergent phage-resistant variants [51]. Additionally, to minimize the potential for the development of resistance, a combination of phages with antibiotics might be required, since there is usually no cross-resistance with antibiotics [52]. Besides, when combined with antibiotics, phages are likely to enhance the absorption of antibiotics into bacterial cells [53–56]. Further study is required to evaluate the effectiveness of the combining phages with antibiotics in the treatment of *A. hydrophila* infection. In this study, we have not detected phage titres in all organs. In this regard, it would be interesting to determine if the phages localised to the high bacterial titre at infection sites.

In aquatic environments, biofilm formation plays important roles in the survival and pathogenicity of pathogens [57]. The extracellular biofilm matrix serves as a scaffold that protects the bacteria encased in the matrix from various harmful environment including antimicrobial agents and cellular host defenses [58]. Biofilm formation is a major barrier to the current treatment method relying on antibiotics. Although there have been several studies on controlling *Aeromonas* spp. using bacteriophages [22, 37, 59], no reports are available regarding phages that can control biofilm formation of *A. hydrophila*. In this study, phages G65 and Y81 showed excellent effect on preventing formation of biofilm at 24 h after coculture even at a small MOI of 0.01, while relatively poor performance was observed in the other three phages. Additionally, almost no bacterial growth was detected at 24 h after treated with phages G65 or Y81. To clear whether the biofilm decrease depends on the phage activity to digest the biofilm or the reduction of bacterial viability, we detected the activities of the five phages on the biofilm formation of *A. hydrophila* XX-58 (accession number of *gyrB* gene: JX025795.1), which was determined to be not susceptible to the infection of the phages in this study. The data indicated that the biofilm formation of XX-58 was not affected by the phage activity (Additional file 1). Our results suggest that the diverse roles of the five phages in preventing biofilm formation were associated with their killing effect on the viable bacterial cells, not with the digestion of the biofilm. Also, we found that the biofilm inhibition of different phages require different MOIs, which is probably owing to the fact that MOI is another major factor determining phage’s ability to adsorb host cells in addition to receptors [60].

The phages G65, W3 and N21 reduced mature biofilm for more than 60% after treating for 24 h. Although phage Y81 showed considerable inhibition effect on biofilm formation, its removal effect on mature biofilm was very low. Interestingly, phages N21 and Y71 behaved similarly in terms of bacteriolytic activities and biofilm inhibiting effect but quite differently in disaggregation of biofilm. As the nutritional and metabolic states as well as phage receptors of bacterial cells in attached state will be different from those in planktonic culture [61], it is not surprising that phages which exhibit poor inhibition effect on bacterial growth and biofilm formation can have high lytic activity to attached bacteria. Although production of structured extracellular polymers of biofilm, in some cases, provides a physical barrier between phages and their receptors, many biofilms have an open structure with fluid-filled channels and pores that would allow the phages access to the bacteria within biofilms [61, 62]. Therefore, we speculate that the different removal efficiency of five phages to the biofilm might be linked to the biofilm structures of *A. hydrophila* NJ-35 and XY-16. Additionally, some phages can even produce polysaccharide depolymerases to degrade the extracellular matrix, allowing phages to come in contact with the encased bacteria [63]. For instance, *Pseudomonas* spp. phage F116 produces an alginate lyase that can reduce the viscosity of the alginate exopolysaccharide and promote phages to penetrate the alginate matrix and reach the bacterial surface [64]. Thus, diverse antibiofilm efficacy of the five phages investigated in this study may also be due to the different activities of enzymes that can degrade exopolysaccharides. In addition, phages N21 and G65 displayed semitransparent halos around phage plaques on the lawns of host bacteria, and halo formation has been proposed to play important roles in exopolysaccharide depolymerization and biofilm degradation [65, 66]. This led us to speculate the halo formation of phage G65 and N21 might be associated with their efficient biofilm removing ability. Nevertheless, this hypothesis remains to be defined in future studies.

## Conclusions

In this study, five *A. hydrophila* phages were isolated, with three belonging to family *Myoviridae* and two belonging to family *Podoviridae* morphologically. All of the five phages have broad host ranges, low letent periods, wide pH and thermal tolerance, and effective treatment of *A. hydrophila* infection. More importantly, several phages exhibited varying abilities to prevent and remove *A. hydrophila* biofilm. This study provides a basis for therapeutic applications of phages to control *A. hydrophila* infection.

## Methods

### Bacterial strains and growth conditions

Two epidemic strains of high virulence isolated from diseased crucian carp, *A. hydrophila* NJ-35 (accession number: CP006870.1) from Nanjing, China in 2010 and XY-16 (accession number of *gyrB* gene: JX025797.1) from Xinyi, China in 2009 [67, 68], were used for phage isolation. An additional 203 *Aeromonas* strains, including 73 *A. hydrophila*, 85 *A. veronii*, 12 *A. caviae*, 1 *A. bestiarum*, 12 *A. sobria*, 10 *A. media*, 3 *A. salmonicida*, 3 *A. jandaei* and 4 *A. aquariorum* were used for host range analysis. All bacterial strains were routinely cultured in Luria Bertani (LB) broth (Difco/Becton Dickinson) at 28 °C with shaking at 180 rpm. All *Aeromonas* strains used in this study are listed in Table 1.

### Phage isolation, purification and propagation

*A. hydrophila* strains NJ-35 and XY-16 were used as indicator hosts for phages. Ten water samples were collected from ponds, sewage and rivers in Nanjing. The samples were centrifuged at 4000 *g* for 30 min and the supernatants were filtered through 0.22-μm membrane filters. A 10 mL of the supernatant was added into 20 mL of 3 × LB broth, and 1 mL cultures of NJ-35 or XY-16 (late logarithmic phase) were used to inoculate the mixture. After adding 5 M CaCl_2_ to a final concentration of 0.1 mM, the phages were enriched by culturing for 14 h at 28 °C. A 3 mL of the culture was added to a 5 mL LB broth, followed by the addition of chloroform with a final concentration of 3%, vigorously shaken for 2 min. After static layering, 1 mL of the supernatant was filtered through a 0.22-μm filter. To confirm the presence of the lytic phage in the fltrate, the double-layer agar method [69] was performed using the filtrate. After incubating at 28 °C for 8 h, a single plaque was picked up with a sterile pipette tip into LB broth with the addition of host bacteria. After proliferation, the phages were purified several times using the double-layer agar method.

### Host range

The host range of the harvested phages was determined using a spot assay. *Aeromonas* strains were spread evenly on the LB agar plate. Five microliters of the phage cultures of 10^8^ PFU/mL were dropped onto the overlaid top agar. After cultured for 12 h at 28 °C, the presence or absence of a lysis zone was observed.

### TEM analysis

The phages were cultured at 28 °C for 8–10 h using the double-layer plates. A plate with plenty of plaques whose edges were faintly visible was added with 2 mL of sterilized double deionized water (ddH_2_O). The plate was shaken horizontally for 5 min to fully wash off the phages. Suspensions in the plate were centrifuged at 6000 *g* for 5 min. For TEM analysis, 15 μL of the phage supernatant was spotted on a carbon/Formvar-coated 200-mesh copper grid (Ted-Pella Inc., CA, USA). After 3 min, the suspension was removed by filter paper and the grid was baked under incandescent light for 5–10 s. The phages were negatively stained with 2% uranyl acetate for 1 min. Excess dye was removed by the filter paper and the grid was dried under an incandescent lamp. The morphology of the phage was imaged by TEM (H-7650, Hitachi, Japan) operated at 80 kV. Phage dimensions were calculated by measuring the dimensions of five independent phages.

### Determination of optimal MOI

The optimal MOI is the ratio of the number of phages to that of host bacteria present in a defined space that is best for phage proliferation to obtain maximum titers. *A. hydrophila* strains grown to the log phase were washed three times with PBS and adjusted to corresponding densities of 10^5^, 10^6^, 10^7^ and 10^8^ CFU/mL, respectively. The phages and bacteria were mixed with MOIs of 100, 10, 1, 0.1, 0.01, 0.001 and 0.0001, respectively. After incubation for 2 h at 28 °C, the phage titers were measured by the double-layer agar method.

### One-step growth curve

One-step growth experiment was performed according to the method described by Adams [43]. *A. hydrophila* NJ-35 and XY-16 were cultured in 1 mL LB broth to OD_600_ of 0.5–0.6 and collected by centrifugation. The cells were resuspended with 0.9 mL fresh LB broth and mixed with 0.1 ml phage solutions (1 × 10^8^ PFU/ml). The phages were allowed to absorb for 5 min and then centrifuged at 13,000 *g* for 1 min to remove free phage particles. After discarding the supernatants, the phage-infected bacterial pellets were resuspended in 50 mL of prewarmed LB broth and the cultures were continuously incubated at 28 °C. At an interval of 5 min, samples were harvested and phage titers were immediately determined by the double-layer agar method. The burst size of phages was calculated by dividing the final titer of released phage particles by the initial count of infected bacterial cells.

### pH and thermal stability assays

For pH stability tests, 100 μL phage suspension (1.0 × 10^7^ PFU/mL) was used to inoculate 900 μL physiological saline adjusted to pH values of 3 ~ 12 with NaOH or HCl. The mixtures were incubated at 28 °C for 2 h and aliquots were taken to measure the titers of phages at different pH values. For thermal stability tests, 2 mL phage suspension (1.0 × 10^7^ PFU/mL) was incubated at 30 °C, 40 °C, 50 °C, and 60 °C. At an interval of 20 min, 100-μL aliquots were collected until 100 min. Survived phages were counted and the survival rates were calculated by the PFU at each time point divided by that at the primary PFU. All tests were performed in triplicate.

### Phage killing assay in vitro

Bacterial killing assays were conducted for the phages as previously described [70]. Briefly, *A. hydrophila* NJ-35 and XY-16 suspensions (1 × 10^8^ CFU/mL) in exponential period were mixed with an equal volume of phages at different MOIs of 0.01, 0.1, 1, and 10, respectively, followed by incubation at 28 °C for 24 h. *A. hydrophila* cultures mixed with LB broth served as positive controls. The absorbances (OD600) of the cultures were measured at each time point. The experiment was performed in triplicate.

### Biofilm formation assay

*A. hydrophila* strains were cultured in LB broth to logarithmic period and then normalized to 1 × 10^6^ CFU/mL. The suspensions were mixed with equal volume of phages with the MOI of 0.01, 0.1, 0, 1, and 10, respectively. Two hundred microliters of the mixtures were added into each well of the 96-well plates. Each treatment was performed in eight replicates. Fresh LB broth served as a blank control. Then the plates were incubated at 28 °C for 24 h. Next, the culture supernatants were discarded and the plates were washed three times with sterile PBS to remove all planktonic cells. Biofilms formed in each well were fixed with 200 μL of 99% (vol/vol) methanol for 15 min. After drying, biofilms were stained with 1% crystal violet for 10 min. Then the wells were washed with distilled water to remove unbound dye. Crystal violet was dissolved in 200 μL of 95% ethanol for 10 min and the absorbance was measured at 595 nm (OD_595_) using a micro-plate reader (Tecan, Switzerland).

### Biofilm clearance assay

*A. hydrophila* strains were cultured in LB medium to logarithmic period and then normalized to OD_600_ of 1.0. The suspensions were inoculated to LB media (200 μL per well) in 96-well plates at a ratio of 1:1000. The plates were incubated at 28 °C for 24 h without shaking. After removing all planktonic cells, each well were treated with 200 μL phage dilution of 1 × 10^8^ PFU/mL with sodium chloride-magnesium sulfate (SM) buffer (100 mM NaCl, 50 mM Tris pH 7.5, and 10 mM MgSO_4_) for 6 h, 12 h, and 24 h. Biofilms treated with SM buffer served as the control. The microplates were then washed twice with PBS and the biofilms left were stained and measured by crystal violet method. The OD_595_ absorbance of each well was measured using a micro-plate reader. The percentage of biofilm left was calculated by dividing OD_595_ of phage treated wells and the OD_595_ of controls without phage treatment multiplied by 100. The percentage was subtracted from 100% in order to get the percentage of biofilm clearance.

### Phage therapy assay in mice model

The animal experiment was performed in accordance with the animal welfare standards, complied with the guidelines of the Experimental Animal Welfare Ethics Committee, Chinese Association for Laboratory Animal Sciences, and was approved by the Ethical Committee for Animal Experiments of Nanjing Agricultural University, China (approval number: SYXK(Su) 2017–0007). Six-week-old female Institute of Cancer Research (ICR) mice (body weight, 18 ± 2 g) were purchased from the Experimental Animal Center of Yangzhou University and raised in the Experimental Animal Center of College of Veterinary Medicine, Nanjin Agricultural University under specific-pathogen-free (SPF) conditions. The dose of *A. hydrophila* used in this study was chosen on the basis of a preliminary study that the mice were expected to show obvious clinical signs but no death. *A. hydrophila* NJ-35 and XY-16 grown to log phase were washed three times with PBS and adjusted to 5 × 10^7^ and 1 × 10^8^ CFU/mL, respectively. For phage therapy assay, a total of 120 mice were randomly divided into three groups and housed in cages (5 mice per cage). Feed and water were allowed ad libitum. To evaluate treatment efficiency of phages in *A. hydrophila* NJ-35 infection, 40 mice were intraperitoneally inoculated with 100 μL of bacterial suspension of *A. hydrophila* NJ-35; after 30 min, the infection group of 10 mice were then intraperitoneally injected with 100 μL of sterile PBS, while each 10 out of the other 30 mice in the treatment group were injected with 100 μL of phages N21, W3 or G65 at the MOI of 1. The blank control group of 30 mice were intraperitoneally injected with 100 μL of sterile PBS, followed by injecting 100 μL of each of the phage suspensions. Similarly, 50 mice were used to determine the therapy effect of phages Y71 and Y81 on *A. hydrophila* XY-16 infection. After 6 h and 24 h, five mice in each treatment were euthanized by CO_2_, respectively. Hearts, livers, spleens, lungs and kidneys were aseptically removed from the mice, weighed, added to 1 mL of sterilized PBS, homogenized using a tissue homogenizer (Bioprep-24, Allsheng, Hangzhou, China), and then 10-fold serially diluted in PBS. Bacterial loads in tissues were determined by plating the dilutions on LB plates. The results are expressed as the numbers of CFU/g tissue.

### Statistical analyses

Data were collected and analyzed using GraphPad Prism version 5 software. A *t*-test was used to analyze the difference between the phage-treatment groups and the control groups. Error bars presented in the figures represent standard deviations of the means of three replicate experiments. A *P*-value < 0.05 was considered as a significant difference.

## Supplementary information

**Additional file 1.** Phage activity on the biofilm formation of *A. hydrophila* XX-58. The biofilm formation ability of *A. hydrophila* XX-58, a bactreiophage-resitant strain, co-incubated with phage N21, W3, G65, Y71 or Y81 at an MOI of 0.01, 0.1, 1 and 10 was measured using crystal violet staining method. Data are shown as the mean ± SD from the triplicate experiments.

## Data Availability

All data generated or analyzed during current study are available from the corresponding author on reasonable request.

## Abbreviations

MDR: Multidrug resistance
UPEC: Uropathogenic *Escherichia coli*
LB: Luria Bertani broth
MOI: Multiplicity of infection
TEM: Transmission electron microscopy
CFU: Colony-forming units
PFU: Plaque forming units
PBS: Phosphate buffer saline
SM: Sodium chloride-magnesium sulfate
SPF: Specific-pathogen-free
ICR: Institute of Cancer Research
